# Psychological Distress and Post-Traumatic Symptoms Following Occupational Accidents

**DOI:** 10.3390/bs3040587

**Published:** 2013-10-25

**Authors:** Marta Ghisi, Caterina Novara, Giulia Buodo, Matthew O. Kimble, Simona Scozzari, Arianna Di Natale, Ezio Sanavio, Daniela Palomba

**Affiliations:** 1 Department of General Psychology, University of Padova, Via Venezia, 8, 35131 Padova, Italy; E-Mails: caterina.novara@unipd.it (C.N.); giulia.buodo@unipd.it (G.B.); scozzari.simona@gmail.com (S.S.); arianna.dinatale@email.it (A.D.N.); ezio.sanavio@unipd.it (E.S.); daniela.palomba@unipd.it (D.P.); 2 Department of Psychology, Middlebury College, McCardell Bicentennial Hall, 276 Bicentennial Way, Middlebury, VT 05753, USA; E-Mail: mkimble@middlebury.edu

**Keywords:** work accidents, trauma, psychological distress, post-traumatic stress disorder, assessment

## Abstract

Depression and post-traumatic stress disorder frequently occur as a consequence of occupational accidents. To date, research has been primarily focused on high-risk workers, such as police officers or firefighters, and has rarely considered individuals whose occupational environment involves the risk of severe, but not necessarily life-threatening, injury. Therefore, the present study was aimed at assessing the psychological consequences of accidents occurring in several occupational settings (e.g., construction and industry). Thirty-eight victims of occupational accidents (injured workers) and 38 gender-, age-, and years of education-matched workers who never experienced a work accident (control group) were recruited. All participants underwent a semi-structured interview administered by a trained psychologist, and then were requested to fill in the questionnaires. Injured workers reported more severe anxious, post-traumatic and depressive symptoms, and poorer coping skills, as compared to controls. In the injured group low levels of resilience predicted post-traumatic symptomatology, whereas the degree of physical injury and the length of time since the accident did not play a predictive role. The results suggest that occupational accidents may result in a disabling psychopathological condition, and that a brief psychological evaluation should be included in the assessment of seriously injured workers.

## 1. Introduction

Work-related accidents occur with some frequency. The Eurostat estimates that about 2.8 million work-related accidents occurred in the European Union in 2009 [1]. To date, studies have primarily investigated the impact of working conditions, or organizational and social contributors to the occurrence of occupational accidents [2,3,4]. Some have evaluated the role of individual characteristics or personality factors in increasing the risk for occupational accidents [5,6]. Others have focused on the physical and social impairment or on the legal consequences [7,8]. 

However, these systematic assessments rarely include an evaluation of the psychological sequelae of occupational accidents [9,10]. This is somewhat surprising given that early work in this area indicates that injured workers show higher rates of depression, anxiety and substance use disorders, compared to the general population [11,12]. Moreover, victims of occupational accidents report pain, inactivity, sleep disorders, intrusive accident memories, impairment in contextual memory and emotional disorders, such as anxiety, depression and irritability [9,13,14,15,16,17]. In addition, many victims of work accidents fulfill the diagnostic criteria for Acute Stress Disorder or Post-Traumatic Stress Disorder (PTSD) [18]. For example, Asmundson and colleagues [9] reported that 34.7% of injured workers with chronic pain achieved full criteria for PTSD, and 18.2% had partial PTSD (*i.e.*, experiencing symptoms in two of the three PTSD symptom clusters, namely re-experiencing, avoidance, and arousal). A more recent study highlighted that six months after the accident 12% of injured workers fulfill criteria for PTSD, with 11% suffering from subclinical PTSD [19]. In other work, the estimated incidence of PTSD in injured workers eight months post-accident is around 18% [20,21].

Most of the studies that assessed the psychological consequences of work-related accidents have focused on specific job categories where ongoing work stress and traumatic events are common, such as police officers, firefighters, emergency service personnel, and paramedics [22,23,24,25]. Few studies have assessed the psychological sequelae of accidents occurring in work populations for which traumatic events may be less expected and frequent but still possible, such as craftsmen, factory workers and laborers. After an accident, these workers show higher level of depressive and anxious symptoms, sleep disturbances, somatic complaints, clinical or subclinical PTSD, and poorer coping skills compared to workers who did not sustain accidents [10,17,26]. Indeed, it should not be surprising that an occupational accident occurring to such workers may also result in the development of PTSD. For these job categories, a work-related accident could represent an unexpected and sudden event, which might involve serious injury, life threat, loss of life to a colleague, or a threat to the physical integrity of self or others. All these possibilities are included in the criterion A of PTSD diagnosis in the current Diagnostic and Statistical Manual of Mental Disorders (DSM-5) [27]. Furthermore, the high levels of depression and anger and poor coping skills found in victims of work accidents may act as vulnerability factors for the development and maintenance of PTSD [28,29,30].

The existing literature suggests that individuals suffering from PTSD are generally more at risk to be unemployed or under-employed [31,32], and those with sub-threshold PTSD are at risk for poor work functioning [33,34]. Injury following occupational accidents may negatively impact motivation to work, job satisfaction, and the cognitive abilities necessary to return to work [35]. In a recent study [36] it has been shown that injured workers, compared to controls, displayed impaired attention and concentration, memory, and executive functions. These cognitive deficits may account for the high rates of re-injury (30% over six months and 16% over one year) detected among victims of a work-related accident [8,37].

The present study was aimed at addressing the following hypotheses: (1) workers that sustained an accident would complain of more severe psychopathological symptoms and, in particular, PTSD, as compared to controls; (2) the severity of post-traumatic symptoms would predict employment status; (3) PTSD severity would be associated with psychological factors (such as resilience, anxiety and anger) rather than accident-related variables (*i.e.*, the severity of physical impairment and the time since the accident).

## 2. Method

### 2.1. Participants

The sample consisted of 38 victims of occupational accidents (injured workers) and 38 workers who never experienced a work accident (control group). 

Injured workers were recruited in several towns in Italy among members of the Associazione Nazionale Mutilati e Invalidi del Lavoro (ANMIL, a non-profit organization supporting individuals who sustained a work accident). The inclusion criteria for participating in this study were as follows: age ranging between 18 and 50 years; competence to give informed consent; length of time since the accident between 6 months and 7 years; degree of physical impairment between 25% and 75%, as assessed by the Istituto Nazionale Assicurazione contro gli Infortuni sul Lavoro (Italian Workers’ Compensation Authority), in order to exclude participants with minor injuries and those with injuries so severe as to impede their participation in the psychological evaluation. Mean degree of impairment was 46.73% (S.D. = 13.5; range 25–75) and the average time since the accident was 5.2 years (S.D. = 1.8; range 1–7). The employment profile of the injured group before the occupational accident was: 21 factory workers, 3 electricians, 3 clerks, 2 drivers, 2 bricklayers, 1 chef, 1 engineer, 1 storekeeper, 1 housepainter, 1 businessman, 1 landscaper, and 1 technician. The type of accidents could be classified as follows (number of participants in parenthesis): amputation of the non-dominant hand or arm (4), amputation of foot or leg (3), being burnt (1), being caught in, under, or between something (11), fall at the same level or from an elevation (7), being struck by or against something (8), and other (4).

The control group was made up of gender-, age-, and years of education-matched participants recruited among friends and acquaintances of the injured workers. Inclusion criteria were the same as for the injured group, except for the absence of work-related accidents.

For both groups, reasons for the exclusion or non-participation were: the presence of physical illnesses and psychopathology unrelated to the work accident; use of drugs or medications that could affect the individual’s ability to perform the assessment; incapacity to give informed consent. For the group of injured workers, additional exclusion criteria were: traumatic brain injury, sensory (visual or hearing) loss, and damage to the dominant hand and/or arm as a consequence of the accident.

The two groups did not differ on marital status (χ^2^ = 2.88; *p* = 0.24), while 39.5% of injured workers *vs.* 2.6% of controls were unemployed (χ^2^ = 15.52; *p* < 0.0001). Student’s t-tests performed on age and years of education revealed no significant differences between groups (see Table 1).

**Table 1:** Mean values ( ± S.D.) of socio-demographic data in injured and control groups.

	Injured workers	Controls
**Age (years)**	36.11 (± 7.34)	35.5 (± 9.42)
**Gender (M/F)**	34/4	34/4
**Education (years)**	11.11 (± 2.82)	12.45 (± 3.5)

### 2.2. Measures

All participants were assessed using the following measures:

*A semi-structured interview* was aimed at collecting socio-demographic data (age, marital status, education, use of medication, presence of physical illnesses). At the beginning of the interview, controls were screened to ascertain they had not experienced any severe traumatic event, by asking if they had ever dealt with a situation, which involved actual or threatened death or serious injury to self or others. For the injured group only, data on the following variables were collected: the degree of physical impairment, the absence of other traumatic events, and a description of the work accident.

*Beck Depression Inventory—II* (BDI-II) [38], Italian version by Ghisi, Flebus, Montano, Sanavio, and Sica [39] is a 21-item self-report scale assessing the severity of depression, with higher scores reflecting more severe depressive symptoms. 

*State-Trait Anxiety Inventory—Y2 Trait Form* (STAI Y2) [40], Italian version by Pedrabissi and Santinello [41] is a 20-item self-report instrument that assesses trait anxiety, with higher scores reflecting higher levels of anxiety.

*State-Trait Anger Expression Inventory* (STAXI) [42], Italian version by Comunian [43] is a 10-item questionnaire addressing the experience and the expression of anger. In the present study only the Trait scale was administered, with higher scores reflecting higher levels of trait anger. 

*PTSD Symptom Scale* (PSS) [44] is a 17-item scale measuring the frequency of PTSD symptoms according to DSM III-R. The PSS is composed by three subscales: re-experiencing, avoidance, and arousal. The total score reflects the severity of PTSD symptomatology. 

*Connor-Davidson Resilience Scale* (CD-RISC) [45] is a 25-item self-report instrument assessing resilience, as a measure of stress coping ability. Example items are “When things look hopeless, I don’t give up”, “Coping with stress strengthens”. Higher scores reflect greater resilience.

The Italian translations of the PSS and the CD-RISC were obtained from back translations by two psychologists who were native English speakers. 

### 2.3. Procedure

The study was conducted in accordance with the Declaration of Helsinki and approved by the institutional board of the participating institution. All individuals gave their written consent before entering the study. Eligible participants underwent the semi-structured interview, administered by a trained psychologist, and then were requested to fill in the questionnaires. The sequence of questionnaires administered to the participants was rotated to control for order effects.

### 2.4. Data Analysis

The level of significance was set at α = 0.05 for all statistical tests. Student’s t-tests were performed to compare self-report data between groups (injured workers *vs.* controls). To further evaluate the magnitude of differences effect sizes were computed. According to Cohen [46] effect size of 0.2–0.3 is considered a small effect, around 0.5 a medium effect and above 0.8 a large effect. Within the injured group, the following analyses were performed: Student’s t-tests (and the respective Cohen’s *d*) to compare self-report data between employed and unemployed participants; Pearson’s product-moment correlation to examine the relationships between post-traumatic symptoms severity and accident variables (length of time since the accident and degree of physical impairment), and other self-report data; forward stepwise (Wald) binary logistic regression analysis to evaluate the role of PTSD symptoms in predicting return to work; block multiple linear regression analyses to verify whether trait psychological variables and accident-related features predict PTSD development.

## 3. Results

Injured workers reported significantly higher levels of anxiety and depression, and lower resilience scores, as compared to controls (see Table 2). The between-group difference in anger scores was nearly significant. Furthermore, in injured workers the mean PSS total score was 19.32 (S.D. = 12.64), indicating moderate severity of PTSD symptoms. Specifically, 39.4% of injured workers in this sample achieved criteria for PTSD based on recommended PSS cut-off scores [47]. For depression, 47.4% of injured workers had a BDI-II score above the cut-off.

**Table 2:** Mean self-report questionnaire scores and differences between groups. Values are mean ± S.D.

	Injured workers	Controls	t	df	*p*	*d*
**BDI-II**	13.95 (± 10.46)	3.71 (± 3.76)	5.68	74	<0.001	1.3
**PSS Total**	19.32 (± 12.64)	1.91 (± 4.22)	7.55	69	<0.001	1.85
**STAI Y2**	42.03 (± 10.48)	32.89 (± 6.81)	4.48	73	<0.001	1.03
**STAXI**	10.50 (± 2.78)	9.21(± 2.88)	1.98	74	=0.05	0.46
**CD-RISC**	61.32 (± 17.33)	74.16 (± 9.99)	−3.96	74	<0.001	−0.91

**Legend**: BDI-II: Beck Depression Inventory—II; PSS Total: PTSD Symptom Scale Total Score; STAI Y2: State-Trait Anxiety Inventory—Y2 Trait Form; STAXI: State-Trait Anger Expression Inventory; CD-RISC: Connor-Davidson Resilience Scale.

Student’s t-tests performed to compare self-report data in employed and unemployed injured workers did not show any significant difference (see Table 3).

**Table 3:** Mean self-report questionnaire scores and differences between employed and unemployed injured workers. Values are mean ± S.D.

	Employed (N = 23)	Unemployed (N = 15)	t	df	p	*d*
**BDI-II**	12.04 (± 10.60)	16.87 (± 9.87)	1.43	36	0.16	−0.47
**PSS Total**	16.39 (± 13.13)	23.80 (± 10.75)	1.90	36	0.07	−0.62
**STAI Y2**	40.39 (± 10.47)	44.71 (± 10.31)	1.23	35	0.23	−0.42
**STAXI**	10.26 (± 2.70)	10.87(± 2.95)	0.64	36	0.53	−0.22
**CD-RISC**	61.70 (± 16.91)	60.73 (± 18.55)	−0.16	36	0.87	0.05

**Legend**: BDI-II: Beck Depression Inventory—II; PSS Total: PTSD Symptom Scale Total Score; STAI Y2: State-Trait Anxiety Inventory—Y2 Trait Form; STAXI: State-Trait Anger Expression Inventory; CD-RISC: Connor-Davidson Resilience Scale.

Forward stepwise (Wald) binary logistic regression analysis performed to assess whether PTSD symptoms (re-experiencing, avoidance, arousal) predicted occupational status in the injured group revealed that only the “Re-experiencing” subscale of the PSS predicted non-return to work (β = −0.18; *p* = 0.04), whereas “Avoidance” (*p* = 0.89) and “Arousal” (*p* = 0.54) subscales were not significant predictors. 

In injured workers, significant positive correlations emerged between PSS total score and BDI-II (r = 0.74), STAI-Y2 (r = 0.66), and STAXI (r = 0.48) scores. Also, there was a significant negative correlation between the PSS total score and the CD-RISC score (r= −0.64). However, no significant correlations were found between the PSS total score, the degree of physical impairment (r = −0.29), and the length of time since accident (r = −0.03).

A series of regression analyses, computed in the injured group to better clarify the previous findings, showed that only low levels of resilience predicted post-traumatic symptomatology. It is important to note that none of the accident-related variables played a predictive role (see Table 4).

**Table 4:** Block multiple linear regression analysis (injured group). Dependent variable: PSS Total. Predictors: accident-related features (time since injury, degree of impairment) and psychological variables (STAI Y2, STAXI, CD-RISC).

	β	Standard Error	t	*p*
**Time since injury**	0.04	0.99	0.28	0.78
**Degree of impairment**	−0.09	0.12	−0.63	0.53
**STAI Y2**	0.23	0.25	1.05	0.30
**STAXI**	0.21	0.79	1.21	0.24
**CD-RISC**	−0.39	0.12	−2.26	<0.05

**Note**: Significance of overall multiple regression: R^2^ = 0.55, F_(5,30)_ = 7.19, *p* < 0.001; **Legend**: STAI Y2: State-Trait Anxiety Inventory—Y2 Trait Form; STAXI: State-Trait Anger Expression Inventory; CD-RISC: Connor-Davidson Resilience Scale.

## 4. Discussion

The present study assessed the severity of psychopathological symptoms in injured workers and their relationship with employment status and accident-related variables (*i.e.*, the severity of physical impairment and the time since the accident). 

As for the psychological sequelae, victims of work-related accidents showed clinically relevant psychopathological symptoms, including post-traumatic symptoms, anxiety, depression, anger, and lower resilience. In the present study, 39.4% of injured workers experienced full PTSD symptoms. This finding is in line with rates reported by Asmundson and colleagues [9] and slightly higher than the rates obtained by Nyberg and colleagues [19] and Matthews [20] who assessed injured workers six to eight months after the accident. It is noteworthy that all of these studies report rates of PTSD after accidents that are higher than the rates after other types of traumatic events. In particular, some studies on combat veterans report rates of PTSD of 14%–15% [48,49]. The high prevalence of PTSD after a work accident could be due to the presence of body injuries in this kind of traumatized individuals. This hypothesis receives empirical support by multiple studies demonstrating that physical injuries following a traumatic event, above and beyond trauma exposure in itself, are a risk factor for PTSD: a greater number of injured trauma survivors develop PTSD compared to non-injured survivors of the same trauma [48,50,51]. The presence of bodily injuries may act as a visual and proprioceptive reminder of the traumatic event, causing intrusive re-experiencing and, therefore, triggering and maintaining post-traumatic symptomatology [52,53,54]. While this is a likely factor that increases PTSD prevalence among injured workers, the higher rates of PTSD in our study are almost certainly due to the fact that all participants who sustained a workplace accident were bodily injured. In addition, they had at least a moderate level of physical disability, whereas in studies on veterans both injured and non-injured survivors have been recruited.

The present study showed that in injured workers the severity of post-traumatic symptomatology was positively correlated with anxiety, anger, and depression, whilst it was negatively correlated with resilience. With regards to the relationship between PTSD and anxiety, our findings are in line with several studies showing that victims of traumatic events with PTSD suffered from more severe symptoms of anxiety, compared to victims of traumatic events without PTSD [17,55,56]. The association between PTSD and depression has also been widely reported in victims of occupational accidents [9,57]. This finding is of particular importance given that the presence of depressive symptoms in individuals with PTSD is a risk factor predicting the chronicity of PTSD [30,58]. As suggested by Shalev and colleagues [59], PTSD and depressive symptoms, although strictly linked, may be considered independent consequences following trauma, that together increase distress and decrease functioning level.

Our results also showed a negative correlation between PTSD severity and resilience in the injured group, suggesting that those with poorer coping skills may be more vulnerable to developing PTSD after an accident. It is also possible that living with injury and PTSD erodes coping capacity over time. However, we found that low levels of resilience uniquely predicted post-traumatic symptomatology, suggesting poorer protection against the development of PTSD or a worse outcome after PTSD [28,60].

The maintenance of PTSD may also be sustained through ongoing issues with anger in injured workers. The relationship found in the present study between PTSD and anger supports such a claim and is in line with the “fear avoidance theory” [61,62]. According to this theory, in some PTSD patients, feelings of anger would allow those with PTSD to avoid intrusive, fear-related thoughts, negative memories, and the associated negative emotions linked to the traumatic event. Therefore, anger would serve as an emotional avoidance strategy, and the avoidance of distressing feelings would hinder emotional processing, which is necessary to overcome PTSD [29].

No significant correlation was found between the severity of post-traumatic symptoms and the length of time since the accident. This finding suggests that some injured workers still suffer from PTSD years after the traumatic event and, hence, that PTSD does not spontaneously remit after a work accident. Similar findings have been reported by Burgess and colleagues [13] in a sample with traumatic work-related hand injury.

There was no significant correlation between the severity of PTSD symptoms and the degree of physical impairment. This result is in line with the findings of several studies on soldiers and motor vehicle accident survivors [51,63,64,65,66,67]. However, in other studies a positive correlation has been found [68,69,70,71,72,73]. These inconsistencies may reflect the complex nature of the relationship between injury and PTSD, which is possibly mediated by neurobiological and psychological mechanisms [52].

Overall, these results suggest that low resilience and the presence of other psychopathological symptoms, rather than the degree of physical injury and the length of time since the accident, should be considered as relatively high risk factors for PTSD in accident survivors. Therefore, the assessment of psychological functioning after a work accident may be just as important in predicting functional impairment as is the assessment of physical injuries. The presence of psychopathology may have serious implications for those trying to return to work after an occupational accident. Indeed, in injured workers, a strong correlation between depression and return to work has been found [74], and among victims of occupational accidents with chronic pain, those characterized by more severe depressive symptoms and affective pain were less likely to return to work [75]. Eventually this relationship may engender a downward spiral, since unemployment may enhance depression levels, further reducing the likelihood to return to work. Recently, Matthews [20] found that injured individuals with PTSD showed higher rates of non-return to work (42%), compared to injured people without PTSD (9%). Our data can only partially support this trend. Indeed, the percentage of unemployment (53.3%) in injured workers with PTSD was higher, but not statistically different, from the percentage (30.4%) in injured workers without PTSD. However, within the injured group, the post-traumatic symptoms of re-experiencing resulted in a prediction of unemployment. This pattern of findings indicates that, in this sample, PTSD status in and of itself was not associated with unemployment but only the re-experiencing cluster.

In conclusion, our results suggest that occupational accidents may result in a PTSD or other disabling psychopathological conditions that should be assessed beyond physical impairment to assign the disability indemnity to job injured victims. 

Some of the strengths of this study include the recruitment of a sample taken from multiple cities in Italy, and the wide array of the participants’ occupational settings and types of workplace accidents. The limitations include the exclusive reliance on self-report measures, the recruitment of a small, non-randomized sample and the preponderance of males. Although it has been well established that men are considerably more likely than women to have an accident or to die at work [1], the preponderance of males in our sample may limit the generalizability of findings to female victims of occupational accidents. Therefore, further studies should consider the gender variable carefully.

With regard to sample characteristics, another shortcoming is the exclusion of injured workers with lower or higher percentage of disability. This latter factor might have weakened our correlations, particularly those between PTSD and injury severity. Furthermore, the injuries sustained by participants varied with respect to visibility, which might have a relevant impact on psychological outcomes. Finally, the proportion of unemployed participants was larger among injured workers than among controls. Therefore, the current study cannot state conclusively that the more severe psychopathology in the group of injured workers is actually due to injury rather than to unemployment. However, this concern is somewhat mitigated by having compared the severity of psychopathology between unemployed and employed injured participants, who did not differ. This result suggests that the increased psychopathology found in the injured group may, in fact, be a consequence of the accident.

In addition, psychopathology in general was associated with injury status but not employment status. This suggests that return to work is no panacea for those who have been previously injured. If psychopathology, like PTSD, results from an injury it is likely to remain even when employment resumes. The implications are that individuals with PTSD are returning or trying to return to work and may be struggling. Evanoff and colleagues [8] reported that 30% of victims of work-related accidents complained of less ability to concentrate after the accident. Similarly, Buodo and colleagues [36] found in injured workers more difficulties in attention and concentration, compared to controls. It is likely that impairment in concentration may contribute to poor job functioning and could possibly increase the risk for re-injury. Therefore, even if workers that have sustained a job injury return to work, they may demonstrate occupational disability, which may be due to the psychological consequences of a work-related accident.

## 5. Conclusions

Even with the above-mentioned limitations, the present study shows that a disabling occupational accident may increase the risk of psychopathology and, in particular, cause depressive, anxious, and PTSD symptoms. However, a longitudinal study including pre-accident data on psychological functioning would be necessary to draw any strong conclusion about causality. Our results clearly call for more studies on the psychological consequences of a physical injury sustained after a workplace accident. When assessing the consequences of a work-related accident, it should be helpful to use several different measures of outcome from multiple data sources [8], and not only the degree of physical impairment. In particular, the introduction of a routine screening for psychopathology after an occupational accident is recommended.

Our findings indicate that it should be necessary to develop early psychological interventions after work accidents, in order to prevent the occurrence of psychopathological symptoms, to strengthen functional coping skills, and to promote higher quality of life and return to work.
